# Participation preferences of health service users in health care decision‐making regarding rehabilitative care in Germany—A cross‐sectional study

**DOI:** 10.1111/hex.13356

**Published:** 2021-09-14

**Authors:** Lisa A. Baumann, Anna L. Brütt

**Affiliations:** ^1^ Department of Health Services Research Carl von Ossietzky University of Oldenburg Oldenburg Germany

**Keywords:** health care decision‐making, health policy, participation preferences, patient and public involvement, rehabilitation

## Abstract

**Background:**

Involving patients and citizens in health care decision‐making is considered increasingly important in Germany. Participatory structures have been implemented, especially in rehabilitative care. However, it is unknown whether and to what extent German patients and citizens want to participate in decisions that exceed their own medical treatment.

**Objective:**

This study aimed to survey participation preferences and associated factors of health service users in decisions regarding rehabilitative care at micro, meso and macro levels.

**Methods:**

A questionnaire was sent to 3872 former rehabilitants. We collected participation preferences using the Control Preference Scale or an adapted form. Possible influencing factors were examined using logistic regression models.

**Results:**

The response rate was 5.7% (*n* = 217). At all decision‐making levels, joint decision‐making was preferred. At the macro level, preferences for actively participating were the highest. Preferences were significantly interrelated between decision‐making levels. At the micro level, an orthopaedic indication significantly decreased the desire for participation compared to psychosomatic indications (odds ratio = 0.44, *p* = .019).

**Discussion:**

Participants wanted to be equally involved in decision‐making as experts. Higher preferences for active participation at the macro level might be due to dissatisfaction with the current health care organisation and lack of trust in politicians. Compared to the general public, our study sample was older (73.3% between 50 and 69 years) and more often chronically ill—factors associated with increased participation preferences in the literature.

**Conclusion:**

Contrary to the identified preferences, participation opportunities in the German health care system are rare. Further research on participation preferences and structures that enable meaningful involvement are needed.

## 1 BACKGROUND

### 1.1 Relevance of public and patient involvement in health care decision‐making

Public and patient involvement (PPI) in health care decisions is increasingly important in Germany.^1^, ^2^, ^3^, ^4^, ^5^, ^6^ Not only is involvement in decisions at the micro level discussed but also involvement at the meso and macro levels. At the micro level, patients can be involved, for example, in decisions regarding their own treatment, the medical agenda or the place of treatment.^7^, ^8^ In our study, we focus on treatment decisions. Meso‐level decisions concern particular geographical regions or health care facilities, whereas macro level decisions concern the whole health care system on a nation, state or province level (e.g., the financing and organisation of the overall service provision).^9^, ^10^ While micro level decisions are referred to as individual, meso and macro level decisions can be summarized under health policy decisions.^11^

The relevant literature on PPI in health care decisions indicates that PPI leads to improved health care. At the micro level, patient participation leads to increased quality of the decision‐making process,^12^ improved patient knowledge^12^, ^13^ and higher patient satisfaction.^13^, ^14^, ^15^ At the meso level, it can result in more patient‐centred care as well as improved care processes and health outcomes of health care facilities.^16^, ^17^, ^18^, ^19^ At the macro level, PPI can ensure patient‐oriented health policy, leading to more patient‐friendly structures and improved service delivery.^17^, ^18^, ^20^, ^21^, ^22^ As the definition of PPI varies in the literature, we define PPI for the purpose of our study as the involvement of health service users in health care decision‐making processes. Health service users include patients who are acute users of health services as well as citizens who are past and potential users of health services.^23^, ^24^, ^25^

### 1.2 Previous research on participation preferences of citizens and patients

When thinking about increasing PPI in health care decisions, the question arises, whether and to what extent patients and citizens want to participate. While evidence on participation preferences at the micro level is increasing,^13^, ^15^, ^26^, ^27^, ^28^, ^29^, ^30^, ^31^, ^32^, ^33^, ^34^ participation preferences in health policy decisions have been less studied. The majority of studies surveying preferences at the micro level indicate that patients prefer a collaborative decision‐making process,^26^, ^28^, ^32^, ^33^, ^34^ while two systematic reviews with a focus on oncological care found that patients prefer a passive decision‐making process.^27^, ^31^ Studies focusing on health policy decision‐making indicate varying preferences, but most conclude that the public prefers a consultative role.^35^, ^36^, ^37^, ^38^, ^39^, ^40^, ^41^, ^42^, ^43^, ^44^, ^45^ The final decision is rather left to physicians,^37^, ^38^, ^39^, ^41^, ^43^, ^44^, ^45^ traditional decision‐makers (e.g., elected officials, experts or politicians),^35^, ^36^, ^37^ a multiprofessional group^40^ or to patients and their families.^43^

So far, participation preferences have been studied either only at the micro level or only at the health policy level, except for Fredriksson et al.,^38^ who emphasized that looking at this together will lead to a deeper understanding of the requirements for PPI in health care decision‐making. However, how participation preferences between different decision‐making levels are interrelated has not been studied yet.

Factors associated with increased desire for participation in health care decisions at all three decision‐making levels include missing trust in the health care system or in physicians^36^, ^38^, ^46^, ^47^ and female sex.^13^, ^32^, ^36^ Rising age is associated with an increased desire for involvement in health policy decisions initially. Only in very old age groups do participation preferences decrease.^36^, ^38^, ^46^ In contrast, at the micro level, younger age is associated with increased preferences for participation.^13^, ^26^, ^27^, ^32^, ^33^ The influence of education on participation preferences at the micro and macro levels is controversial—in some studies, higher education and in other studies lower education led to increased participation preferences. At the micro level, it is further suggested that participation preferences vary between indications and disease patterns.^13^, ^34^ For health policy decisions, it is emphasized that participation preferences can vary between countries and care settings due to different democratic understanding and culture or the organisation of health care. Therefore, considering the context while assessing participation preferences is important.^38^, ^41^

### 1.3 Participation of citizens and patients in the German setting of rehabilitation

The opportunities for citizens and patients to participate in decision‐making processes differ within the German health care system. In rehabilitative care, participatory structures are already further developed compared to other health care settings.^48^, ^49^, ^50^, ^51^, ^52^, ^53^ This can be seen, for example, in its unique legal anchoring: Rehabilitants should be involved in their own treatment but also in the organization and evaluation of rehabilitative services (German Social Code IX). Different approaches for PPI have been implemented as a result (e.g., patient involvement in quality assurance or development of therapy standards).^48^ However, to implement participation opportunities that correspond to the patients' and citizens' preferences and are perceived accordingly by them, these preferences must be known.

Studies from Germany that have investigated participation preferences of patients and citizens in rehabilitative care focus mainly on micro‐level decisions.^52^, ^54^, ^55^, ^56^, ^57^ Most have found high preferences for participation in treatment decisions,^52^, ^54^, ^56^, ^57^ except for the study of Quaschning et al.,^55^ which indicates varying preferences. The studies were conducted in the inpatient rehabilitation setting and included patients with different indications. Either a cross‐sectional^54^, ^55^, ^56^ or a mixed‐methods design^52^, ^57^ was used, and participation preferences were surveyed using standardized instruments (German version of the Perceived Involvement in Care Scales, the Cologne patient questionnaire, the 9‐item Shared Decision‐Making Questionnaire)^54^, ^55^, ^56^ or self developed instruments.^52^, ^57^

We identified only one study that assessed participation preferences in rehabilitative care at the macro level (by one self‐developed question).^6^ Seventy percent of the included 50 patients from an inpatient cardiac rehabilitation site wanted to be involved in decisions concerning the financing of health services. Studies on participation preferences in overall health policy indicate that German citizens do not see their interests well represented and that they see a need for greater involvement of citizen and patient representatives.^58^, ^59^, ^60^

### 1.4 Study rationale

Studies concerning the individual participation preferences of health service users in rehabilitative care, especially at the health policy level, have rarely been carried out in Germany. Previous results of studies on participation preferences conducted in different health care settings or countries are only limited transferable as the context differ. This study therefore aims to assess the participation preferences of health service users in health care decision‐making at the micro, meso and macro levels in the German setting of rehabilitative care. Additionally, we will examine the impact of sociodemographic factors on, the indication for and treatment satisfaction with these preferences. Considering all three decision‐making levels allows us to understand whether participation preferences and the factors influencing them differ between decision‐making levels and how participation preferences are interrelated. The results can support the discussion on appropriate interventions to strengthen PPI in health care decision‐making.

## 2 METHODS

### 2.1 Study sample and data collection

To determine participation preferences, we conducted a cross‐sectional survey of health service users who previously received rehabilitative treatment in three inpatient rehabilitation centres of the German Pension Insurance Oldenburg‐Bremen between August and December 2020. The study was approved by the responsible Ethics Board (number 2019‐150). Persons were eligible for inclusion if they had completed psychosomatic or orthopaedic rehabilitation at one of the three facilities in 2019 (the discharge report was available). Normally, patients stay in a rehabilitation centre for approximately 3 (orthopaedic) or 6 (psychosomatic) weeks. Due to their recent rehabilitation stay, study participants had individual experiences as patients, but could also take the broader public perspective as they had already completed their inpatient rehabilitation treatment.

The main diagnoses treated in the rehabilitation centres are depression, burnout, personality and behavioural disorders or anxiety disorder for psychosomatic rehabilitation and diseases of the musculoskeletal system, related chronic pain and psychosomatic comorbidities for orthopaedic rehabilitation. A survey questionnaire was sent out via post to 3872 former rehabilitants. The participants could decide whether they wanted to fill out the online survey or the paper‐based survey.

#### 2.1.1 Survey

Our survey was embedded in a larger study on action and research need in rehabilitative care from the viewpoints of rehabilitants and people working in rehabilitative care. The questionnaire consisted of three questions regarding participation preferences in decisions on rehabilitative care at the micro, meso and macro levels (Table 1). Additionally, questions about sociodemographic data, the type of indication and satisfaction with one's own rehabilitation were included.

**Table 1:** Survey questions

Q1 (micro)	First, we would like to know to what extent you want to be involved in decisions concerning your own rehabilitative treatment. Consider a situation with various treatment possibilities that may involve different health outcomes and associated risks. How would you like a decision to be made?
Q2 (meso)	Now we would like to know to what extent you want to be involved in decisions concerning the general organization of rehabilitative treatment. Imagine that a rehabilitation clinic wants to change its services for all rehabilitation patients in the future. For example, this could be the development of new treatment options or it could affect the rehabilitation process. To what extent would you like to be involved in such decisions?
Q3 (macro)	Finally, we would like to know to what extent you would like to be involved in political decisions concerning the design and financing of the rehabilitation system. Imagine that a law that aims to redesign rehabilitative care is to be discussed and passed. Which of the following answer options meets your participation preferences?

### 2.2 Survey of the main outcome variable

Participation preferences at the micro level were measured using the standardized and validated *Control Preferences Scale (CPS)* (Q1).^61^ The *CPS* is considered a reliable tool to measure preferences in health care decisions and is frequently used in the literature for this purpose.^26^, ^28^, ^34^ It measures one aspect of health care decision‐making at the micro level: The level of control that an individual would like to have over decisions concerning his or her medical treatment. The original questionnaire consists of five cards, each with a statement and a related cartoon. The statements represent the different levels of potential participation preferences in treatment decisions. These range from treatment decisions solely made by the patient, a joint treatment decision between the patient and the physician, to the sole treatment decision by the physician. Accordingly, the desire of the patient to decide on his or her treatment can equally be classified as active, joint or passive.^61^

To capture participation preferences at the meso and macro levels, the wording of the scale items and the cartoons were adapted to the new context in consultation with the authors of the *CPS*. The participants were asked, how much control they would like to have over decisions regarding the local organization of rehabilitative treatment (e.g., offer of different treatment options or organization of treatment processes in rehabilitation facilities) (Q2) and over decisions concerning the design and financing of rehabilitation services (Q3). Additionally, each question contained a corresponding practical example from rehabilitation care (Table 1).

For the adapted scales, we assessed psychometric properties. To ensure content validity, we chose similar wording for the questions, answers and cartoons to the original CPS and discussed the items within our study group and with the authors of the CPS. The wording of the items was closely aligned with the definition of the constructs to measure. The questionnaire was then pretested with eight rehabilitants, who were asked to describe how they interpret the items. The pretests showed that participants understood the items and the theoretical constructs behind it. We further assessed criterion and construct validity by comparing our results with other empirical studies that measured the same construct using different instruments. Our results for the adjusted scales were partly inconsistent with the results of previous studies, but this can be explained by the characteristics of our study sample. Hypotheses related to participation preferences in health policy decisions already led us to expect slightly divergent results (see Sections Methods, 3 and Results, 4).

For the assessment of the reliability of the adapted scales, methods common to multi‐item scales were not applicable as the CPS is a single‐item scale. To ensure the reliability of the scales and to avoid misinterpretation, we used easy‐to‐understand question and answer options. The examples for each question further increased the overall comprehensibility of the items.

**Figure 1:**
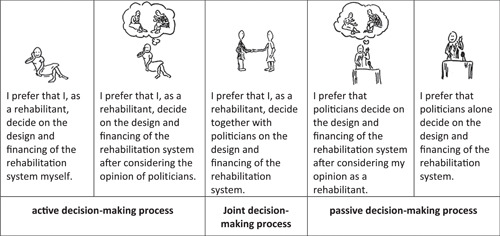
Adapted Control Preferences Scale for health care decisions at the macro level based on Degner et al.^61^

The adapted version of the CPS for macro level decisions is shown in Figure 1. To achieve a sufficiently large number of responses in each answer category, we used the three merged categories active, joint or passive for some statistical analyses (see Figure 1).

### 2.3 Survey of study participants' characteristics

In addition to participation preferences, we obtained information on age, education, gender, indication, years with disease, time period since last rehabilitation and satisfaction with one's own rehabilitation in the survey. Satisfaction with one's own rehabilitation was measured using a five‐point Likert‐type item. All the included variables were assessed in categories. We combined variable categories for some statistical analyses here as well. Age was divided into four categories (*18–39, 40–49, 50*–*59 and 60*–*69 years)*, and education was divided into three categories (*without school‐leaving qualification* or *other*, *medium level of education* [Secondary School Diploma] and *high level of education* [including Technical Baccalaureate/High School Diploma and University of Applied Sciences/University]). The variable indication had three levels (*orthopaedic, psychosomatic* or *both*, when participants stated that they participated in orthopaedic and psychosomatic rehabilitation) as well as satisfaction with their own rehabilitation (*very satisfied* and *satisfied*, *neither nor* and *unsatisfied* or *very unsatisfied)*.

#### 2.3.1 Patient involvement

Former rehabilitants were involved in the development of study documents to ensure comprehensibility. The paper‐based questionnaire was pretested face to face and the online version was completed by four rehabilitants each. Afterward, the questionnaire and study information were revised according to their remarks.

#### 2.3.2 Statistical analysis

We used the statistical software *R* (Version 4.0.3) and *SPSS* (Version 26) for all statistical analyses. We calculated frequency values for participation preferences on each decision‐making level. To understand which combinations of participation preferences across the decision‐making levels are frequently chosen, we created a decision tree.

Before further analyses, we imputed missing data using the fully conditional specification method (MICE). MICE is recommended for data sets containing variables of different types and allowed us to take the uncertainty about the imputed value into account by imputing multiple times. Therefore, parameter estimations are less biased.^62^, ^63^ We assumed that our missing data are missing at random. Overall, we created 40 imputed data sets as recommended by Azur et al.^62^ For imputation, we used the “mice”—package in *R*.^64^

To assess the possible influencing factors on participation preferences, we ran an ordered logistic regression model on each of the imputed data sets. As independent variables, we considered age, gender, education and the indication, as these are mentioned as important predictors in the literature (see Section Background, 1). Furthermore, we considered satisfaction with one's own rehabilitation as we assumed that this is related to trust in the physician and could therefore be an important predictor. We set the significance level for the regression analyses to a two‐sided *p* value of less than .05. We pooled the results of the regression models to one outcome set and calculated odds ratios for the participation preferences depending on the variation of the independent variables.

To assess differences in the distribution of preferences between decision‐making levels, we conducted a Friedman test, followed by a Nemenyi post‐hoc test for pairwise comparisons. To check whether the preferences for an active, joint or passive decision‐making process are correlated between different levels, we conducted a *χ* ^2^ test of independence. When there was a significant correlation, we calculated the Spearman's rank correlation coefficient to assess the strength of the correlation. The significance level was set to a two‐sided *p* value of less than .05.

For the statistical analyses, we had to exclude one case, where the answer for gender was *diverse*, as we had only one person in this category. A statistical analyses was, therefore, not reasonable. We tested, however, whether the assignment of this person to the group *female* or *male* would lead to a significant difference in the results.

## 3 RESULTS

Of the 3872 former rehabilitants contacted, 90 could not be reached. A total of 217 persons participated in our study (response rate 5.7%). Slightly more than half of the participants were male (52.1%). The majority were between 50 and 59 years old (53.5%), had a secondary school diploma (70.5%) and had participated in orthopaedic rehabilitation (57.6%). An overall overview of the characteristics of the study participants is shown in Table 2. For the variables age, gender and indication, we had information on all invited participants. A total of 57.2% were male (43.8% female) and 68.4% had participated in orthopaedic rehabilitation (31.6% in psychosomatic). The majority of the invited participants (46.8%) were between 50 and 59 years old (proportion in other age groups: 18–29 years: 2.5%; 30–39 years: 6.7%; 40–49 years: 18.6%; 60–69 years: 25.4%). While comparing our study sample with the overall study population for these variables, our sample differs only slightly from the overall study population.

**Table 2:** Characteristics of the study participants

Variable	Specification		Missing
Gender	Male	52.1%	2.3%
	Female	45.2%	
	Diverse	0.5%	
Age	18–29	1.4%	2.3%
	30–39	6.9%	
	40–49	16.1%	
	50–59	53.5%	
	60–69	19.8%	
Education	Without school‐leaving qualification	1.4%	2.8%
	Secondary school diploma	70.5%	
	Technical Baccalaureate/High School Diploma	15.2%	
	University of Applied Sciences/University	6.5%	
	Other	3.7%	
Indication	Orthopaedic	57.6%	1.8%
	Psychosomatic	33.2%	
	Both	7.4%	
Satisfaction with own rehabilitation	Very satisfied	29.0%	3.2%
	Satisfied	48.8%	
	Neither nor	7.8%	
	Unsatisfied	6.9%	
	Very unsatisfied	4.1%	
Years with disease	Less than a year	2.3%	3.7%
	1–10 years	48.0%	
	11 years or longer	46.1%	
Time period since last rehabilitation	1–3 months	4.6%	4.1%
	4–6 months	4.1%	
	7–9 months	10.6%	
	10–12 months	31.3%	
	More than 12 months	45.2%	

### 3.1 Descriptive results

**Figure 2:**
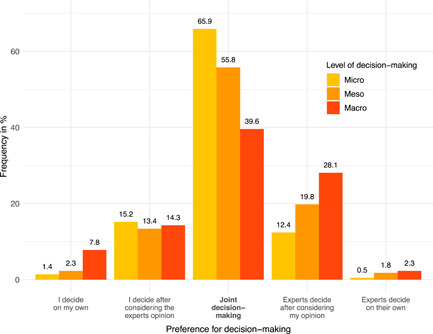
Preference for joint decision‐making at all three levels of health care decision‐making (missing data not shown in the graphic)

The participation preferences at all three decision‐making levels are shown in Figure 2. The greatest desire for a joint decision‐making can be found at the micro level (65.9%). An active form of participation at the micro level is further desired slightly more often (16.6%) than a passive form of participation (12.9%). At the meso level, an active form of participation is desired slightly less often (15.7%) than a passive form of participation (21.6%). The distribution of participation preferences at the macro level is more widely spread. Most participants still preferred joint decision‐making (39.6%), but more people would like to be actively involved in the decision‐making process (22.1%) compared to the micro and meso levels. However, a passive form of participation is also more frequently chosen (30.4%).

**Figure 3:**
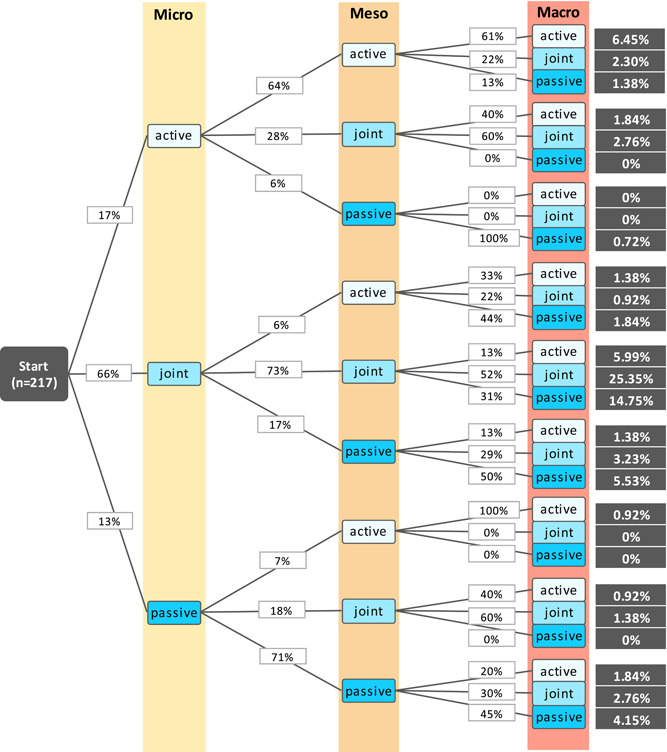
Combination of participation preferences with the probability of being chosen by the study participants (missing data not shown in the graphic)

In Figure 3, it becomes apparent which combinations of participation preferences across the decision‐making levels are most frequently chosen. The combination of a joint decision‐making at all levels is most frequently chosen (25.4%), followed by a joint decision‐making at the micro and meso levels in combination with a passive decision‐making at the macro level (14.8%).

#### 3.1.1 Differences and interrelationships in participation preferences between decision‐making levels

The variation in the distribution of participation preferences between decision‐making levels is slightly significant (*χ* ^2^ = 7.30, *p* = .026). In post‐hoc pairwise comparisons, this significant difference could not be verified.

Between the participation preferences at the different decision‐making levels, we identified significant correlations (*p* < .001 for all combinations of decision‐making levels, see Table 3). We found a large positive correlation between participation preferences at the micro and meso levels (*r*_s_ = .55, *p* < .001), a medium positive correlation between preferences at the meso and macro levels (*r*_s_ = .34, *p* < .001) and a small positive correlation between preferences at the micro and macro levels (*r*_s_ = .21, *p* < .01).^65^

**Table 3:** Statistical outcomes for correlations between participation preferences

	*χ* ^2^	*p* Value	Spearman's rho	*p* Value
Micro–Meso	100.0202	9.739489e−21***	0.55 (CI: 0.44–0.64)	5.42503e−17***
Meso–Macro	36.56588	2.213061e−07***	0.34 (CI: 0.21–0.46)	1.93967e−06***
Micro–Macro	23.54933	9.832825e−05***	0.21 (CI: 0.07–0.34)	.004002325**

Abbreviation: CI, confidence interval.

** *p* < .01.

*** *p* < .001.

#### 3.1.2 Influencing factors on participation preferences

At the micro level, we identified that participants with an orthopaedic indication are significantly less likely to want to be involved in individual treatment decisions compared to those with a psychosomatic indication (*p* = .019). At the meso and macro levels, we did not observe this correlation. We also did not find any influence on participation preferences regarding gender, age, education or satisfaction with one's own rehabilitation. The results of the regression analyses are shown in Table 4.

**Table 4:** Results of ordered logistic regression models

	Micro level	Meso level	Macro level
Independent variables	OR (95% CI)	OR (95% CI)	OR (95% CI)
Gender (reference: male)			
Female	1.34 (0.74–2.44)	1.10 (0.63–1.91)	0.90 (0.52–1.55)
Age (reference: 18–39)			
40–49	0.75 (0.21–2.72)	0.34 (0.10–1.13)	0.81 (0.26–2.53)
50–59	0.96 (0.31–3.01)	0.62 (0.22–1.79)	0.67 (0.24–1.82)
60–69	0.92 (0.27–3.14)	0.82 (0.26–2.53)	0.87 (0.30–2.60)
Education (reference: high level)			
Without educational attainment/other	0.78 (0.18–3.32)	0.49 (0.11–2.21)	0.50 (0.13–2.00)
Medium level	1.36 (0.64–2.91)	0.75 (0.37–1.51)	1.30 (0.67–2.54)
Satisfaction (reference: very satisfied/satisfied)			
Unsatisfied/very unsatisfied	1.13 (0.41–3.13)	1.58 (0.56–4.48)	0.73 (0.30–1.78)
Neither nor	1.39 (0.44–4.36)	1.58 (0.60–4.12)	1.00 (0.35–2.84)
Indication (reference: psychosomatic)			
Orthopaedic	**0.44** * **(0.23–0.87)**	0.82 (0.44–1.52)	0.87 (0.49–1.55)
Both	0.71 (0.22–2.33)	0.87 (0.27–2.76)	1.90 (0.68–5.30)

Abbreviations: CI, confidence interval; OR, odds ratio.

* *p* < .05.

## 4 DISCUSSION

In this study, we identified the participation preferences of health service users in decisions regarding rehabilitative care at the micro, meso and macro levels as well as associated factors with these preferences. Our findings indicate that study participants prefer to be equally involved as experts in decision‐making. At the very least, they want their interests to be heard and considered, even when preferring to not actively make the final decision themselves.

In the following, we discuss participation preferences at the individual decision‐making levels, existing differences and interrelationships between these levels and influencing factors on participation preferences. Finally, we discuss the practical implications of our findings.

### 4.1 Participation preferences at the micro level

The desire for a joint decision‐making process was strongest at the micro level, and simultaneously, we found the strongest rejection of a passive decision‐making process here. This suggests that it is most important for study participants to be equally involved in decisions regarding their individual treatment. Our study findings support existing evidence for a collaborative decision‐making process being preferred by patients.^28^, ^32^, ^33^, ^34^ Other studies from Germany further identified a high participation preference among rehabilitants in treatment decisions.^54^, ^56^

### 4.2 Participation preferences at the meso level

At the meso level, our study findings indicate a stronger desire to be involved in the decision‐making process compared to previous studies, which conclude that a consultative role is preferred.^39^, ^40^, ^41^, ^42^, ^45^ This difference may be due to the setting of rehabilitation. As mentioned in the Section Background, 1, the principle of participation is already widly implemented in some parts of this care setting.^48^, ^49^ Furthermore, rehabilitants must request their rehabilitation stay themselves, so they already need to be actively concerned with their own health care. Study participants may therefore be more familiar with participating in health care decisions. Our study participants were also older and mostly chronically ill, which is typical for rehabilitants and associated with an increased desire for involvement in health policy decisions.^36^, ^38^, ^46^, ^66^ Fredriksson et al.^38^ assumed that this is because of the more frequent contact with the health care system and personal concern. Therefore, compared to the general public, participation preferences for rehabilitants might be higher.

### 4.3 Participation preferences at the macro level

As for the meso level, we identified higher participation preferences at the macro level compared to other study findings.^35^, ^36^, ^37^, ^38^, ^39^, ^40^, ^44^ The setting and the characteristics of the study participants can serve as possible explanatory factors here as well. Surprisingly, we found the highest preferences not only for an active but also for a passive form of participation at the macro level.

The wider spread of participation preferences at this level can be an indicator that the overall interest in policy varies between individuals—some of our study participants might be very interested in general policy issues and prefer active participation, while others might be not interested at all and, therefore, chose a passive form of participation. Missing confidence in politicians to make relevant health care decisions might have further increased the preference for active participation. International studies indicate that the public does not see a legitimate role for politicians as central decision‐makers in health care.^40^, ^43^ A German study showed that the public has little trust that policy decisions at the macro level do not negatively impact patient care.^67^

Dissatisfaction with the current organization of rehabilitative care might also explain the higher preference of actively participating in macro‐level decisions.^38^ As mentioned in Section Background, 1, the majority of the German population feels that their interests are not well represented in health policy, indicating a need for greater PPI from their viewpoint.^58^, ^59^, ^68^ The lack of direct personal concern at this level, on the other hand, could lead to the preference of a passive form of participation. Decisions at the macro level are mostly made from the public perspective and the benefits for the individual are not always so obvious to those involved in the decision‐making process.

The wider spread of participation preferences at the macro level needs to be considered when implementing participative structures at this level. It can lead to a bias in the selection of participants for a macro level decision‐making process, where only the very motivated and political educated individuals participate. Methods that ensure representativeness of participants are therefore necessary.

### 4.4 Differences and interrelationships in participation preferences between decision‐making levels

The results indicate that participation preferences are not equally distributed across the decision‐making levels. As the Friedman test was just significant (*p* = .026) and we could not identify any significant difference for pairwise comparisons in the more conservative post‐hoc analyses,^69^ it should nevertheless be interpreted with caution.

Between the decision‐making levels, participation preferences were significantly positively interrelated at all three decision‐making levels. This allows us to conclude that individuals are likely to have the same participation preferences across decision‐making levels. Based on this knowledge, PPI in health policy decision‐making could be promoted from the micro level, where participation opportunities are already more developed. Since most citizens have contact with the health system during their individual treatment, they can already be empowered and supported here to participate in health care decisions. When patients make positive experiences with participating in micro‐level decision‐making and feel, that their opinion truly matters in the decision‐making process, they might also be willing to participate in decisions at the meso and macro levels.

### 4.5 Influencing factors on participation preferences

We found no influence of age, gender or education on participation preferences at all three decision‐making levels, while other studies did. This might be explained by the socio‐economic characteristic of the study participants. Our study sample was very homogeneous regarding age and education and the biggest part was represented by one or two variable categories. This characteristic is not surprising for the setting,^54^, ^56^, ^66^ but could lead to larger standard errors, and makes it difficult to identify differences between categories. This problem may be solved by a larger sample size in further research. Additionally, age was only available as a categorical variable. This leads to a loss of information in regression analyses and, therefore, significant differences within the categories may not have been identified.

The indication might be an important factor for participation preferences in individual treatment decisions as we found a significant association between an orthopaedic indication and a decreased desire for involvement compared to a psychosomatic indication. That participation preferences at the micro level can vary between indications was already confirmed in the systematic reviews of Tariman et al. and Ernst et al.^13^, ^34^ That the preference for greater participation in decisions redarding one's own medical treatment is more pronounced for psychosomatic patients could lie in the treatment practice in psychiatric care. Here, treatment decisions are often made under the exclusion of patients and associated with coercion.^70^, ^71^ This may have shaped patients and strengthened the desire for active participation.

Since we have only examined those influencing factors that have already been proven to be relevant predictors of participation preferences in the literature, we might have overlooked other crucial predictors. It is conceivable, for example, that participatory structures already implemented in practice and associated barriers (e.g., high time commitment) have an impact on theoretical participation preferences. Further research is necessary to assess additional possible predictors for participation preferences.

### 4.6 Practical implications

As PPI in health care decision‐making is related to improved health systems and patient‐oriented care^12^, ^13^, ^14^, ^15^, ^16^, ^17^, ^18^, ^19^, ^20^, ^21^, ^22^ and the findings of the study indicate that patients and citizens would like to be involved in decision‐making processes, it would be important to implement opportunities for participation at all decision‐making levels. While the relevance attributed to PPI in health policy is increasing, actual participation options are rare in Germany.^4^, ^49^, ^54^, ^72^, ^73^ Although some opportunities for PPI in decision‐making already exist within the setting of rehabilitative care (see Section Background, 1), most of the health policy decisions (e.g., the organization or financing of rehabilitative care) are made within the health care system as a whole and not within individual health care settings. Therefore, the possibilities for patients and citizens to become involved in health policy decisions regarding rehabilitative care are closely linked to the participatory structures in the overall health care system. These are primarily characterized by indirect involvement via patient representatives, who do not have a right to vote but to give advice in important decision‐making processes (e.g., patient representatives in the decision‐making bodies of the Federal Joint Committee [G‐BA] or health care providers). There are also some direct participation opportunities such as social elections, where citizens have the right to elect representatives to the supervisory board of health insurance funds. However, as the number of candidates usually does not exceed the number of seats to be allocated, this can be described as tokenistic involvement.^74^, ^75^ The central decision‐makers in the German health care system are still health care providers and financers. These groups are, compared to health service users, a more homogeneous group with very precise interests and participation preferences. Furthermore, they have significantly more knowledge in the area of health policy.^76^ Therefore, political action is necessary to implement real opportunities for PPI in health policy decision‐making and to redistribute decision‐making power between patients/citizens and health care professionals and politicians.^23^, ^77^ Such participation opportunities must consider heterogeneous interest, participation preferences and capacities (e.g., time capacities) of the public. Different participation opportunities are needed and possible barriers must be dismantled to ensure a representative and legitimate decision‐making process. Before implementing opportunities for participation across the health care system, a representative assessment of participation preferences in health policy decisions of the German population would be necessary. Afterward, suitable PPI methods can be tested, implemented and evaluated.

#### 4.6.1 Limitations

One major limitation of this study is the low response rate and, therefore, the small sample size. A low response rate in this study area is not unusual.^35^, ^39^ However, it restricts the generalizability of our findings. The low response rate may be an indicator of the lack of interest of the general population in participating in health care decisions. Possibly, only the very motivated and interested individuals participated in our study and we, therefore, overestimate the participation preferences. As our study sample was still representative for all invited participants in terms of sociodemographic characteristics, we believe that our results nevertheless add valuable information to the discussion on PPI in health care decision‐making.

The generalizability of our results is further limited by some additional points. We only surveyed former rehabilitants with orthopaedic or psychosomatic indications, so our results may not be transferrable to other rehabilitation settings or the general German population. Moreover, we only included individuals who are insured with the Pension Insurance Oldenburg‐Bremen. The limitation to a specific geographical region could have impacted our results.^30^

Our results may also have been influenced by the quantitative questionnaire format, which could lead to different participation preferences than qualitative methods, where the option for discussion and clarification of questions exists.^39^, ^41^, ^43^ We used closed‐ended questions as we expected a higher response rate,^78^ to include a larger sample size and because of the complexity of the topic. We believe that close‐ended questions are easier to understand and answer for participants. A qualitative research approach to gain insight into the reasons for different participation preferences would be interesting for further research.

We also recognized that the CPS is criticized in the literature for only measuring one aspect of decision‐making at the micro level when focusing exclusively on treatment decisions.^7^, ^8^ More options of decision‐making exist at the micro level and ignoring them might lead to inaccurate assumptions on participation preferences. This problem is also inherent in other comparable scales. Since the CPS proved to be a reliable and easy‐to‐understand instrument, was easily adaptable for the meso and macro levels due to its single‐item characteristic and scale values can be used for ordinal regression analyses, we decided that the CPS was a suitable instrument for our purpose. As we used the adapted versions of the CPS for the first time, further validation would be necessary.

## 5 CONCLUSION

The majority of the study participants wanted to be equally involved like experts at all health care decision‐making levels (micro: 65.9%, meso: 55.8%, macro: 39.6%), regardless of age, gender, education or satisfaction with the received rehabilitative treatment. At the micro level, the patients' indication influenced their preferences.

Contrary to the identified preferences, PPI in health policy decisions in Germany is in its infancy.^4^, ^49^, ^54^, ^72^, ^73^ Health care providers and financers are still the central decision‐makers. The successful implementation of PPI depends on the willingness of policy‐makers to redistribute decision‐making power and on the motivation of service providers to meaningfully involve patients and citizens. It also depends on the motivation of patients and citizens to become involved. As participation preferences between decision‐making levels were significantly correlated, patients can already be motivated and empowered to participate in health care decision‐making at the micro level. This might be a good place to start fostering PPI also in health policy decisions.

To implement appropriate methods, research on the participation preferences of the general German population is needed. Further, it needs to be investigated how citizens and patients would like to become involved and what they need to be able to participate. It would also be interesting to assess why people want to or do not want to become involved. A qualitative research approach could provide valuable information here. Based on this evidence, the implementation of PPI and increased patient‐centredness of the German health care system can be pushed further ahead.

## CONFLICT OF INTERESTS

The authors declare that there are no conflict of interests.

## AUTHOR CONTRIBUTIONS

Anna L. Brütt contributed to the development of the study design. Lisa A. Baumann and Anna L. Brütt contributed to the development of the survey questionnaire. Lisa A. Baumann performed the statistical analyses and wrote the manuscript. Anna L. Brütt supervised the process of manuscript preparation and edited the manuscript. Both authors approved the final version of the manuscript. Rehabilitants and caregivers were involved in the questionnaire design.

## Data Availability

The data that support the findings of this study are available from the corresponding author upon reasonable request.
